# The relationship between in-group favoritism and online prosocial behaviors among college students: the role of moral self and the belief in a just world

**DOI:** 10.3389/fpsyg.2026.1704571

**Published:** 2026-03-27

**Authors:** Keke Zhang, Cheng Chen, Qingsong Sang

**Affiliations:** College of Educational Science, Anhui Normal University, Wuhu, Anhui, China

**Keywords:** belief in a just world, college students, in-group favoritism, moral self, online prosocial behavior

## Abstract

**Objective:**

This study aims to explore the joint mechanism of in-group favoritism and belief in a just world on individual online prosocial behaviors among college students in the context of online donation, with a focus on the mediating role of the moral self and the moderating role of belief in a just world.

**Method:**

A online donation scenario was created through experimental methods to manipulate participants' in-group favoritism (high vs. low) and belief in a just world (priming group vs. control group). Their moral self-level and online prosocial behavior (using comment word count as an indicator of expressive prosocial behavior) were measured.

**Results:**

(1) In-group favoritism significantly and positively predicted online prosocial behavior; (2) The moral self played a significant mediating role between in-group favoritism and online prosocial behavior; (3) Belief in a just world moderated the influence of in-group favoritism on the moral self. Under the condition of activated belief in a just world, the positive predictive effect of in-group favoritism on the moral self was stronger, thereby indirectly affecting prosocial behavior through the moral self.

**Conclusion:**

The present study found that the moral self serves as an internal psychological mechanism through which in-group favoritism influences online prosocial behavior, and that belief in a just world moderates this mechanism. These findings provide a new theoretical perspective for understanding the driving factors of prosocial behavior in online contexts and offer practical implications for guiding college students' online prosocial behavior.

## 1 Introduction

A core tenet of Social Identity Theory is that individuals derive a part of their self-concept from their group memberships. This leads to in-group favoritism—a systematic tendency to favor one's own group through more positive evaluations, affective preference, and behavioral support (Tajfel and Turner, 1979). As a key behavioral manifestation of social identity, in-group favoritism serves to establish and reinforce a collective “we” identity, fulfilling fundamental needs for a positive self-concept and self-esteem (Brewer, 1999; Hewstone et al., 2002; Huang et al., 2016).

The digital landscape offers a potent context for the expression of this tendency. Online environments are characterized by sharpened group boundaries (e.g., explicit community tags) (Zuo et al., 2019), interaction unbound by time and space, and heightened public visibility of actions (e.g., public likes, acknowledgments) (Garcia and Rimé, 2019). These features amplify and reshape how in-group favoritism is expressed, with one critical manifestation being online prosocial behavior—voluntary actions intended to benefit others or society within digital contexts.

According to Social Identity Theory, engaging in prosocial acts toward fellow in-group members serves to bolster self-worth and uphold a positive group image (Bai et al., 2024; Deng et al., 2023). Thus, we posit that group identification acts as the pivotal psychological bridge linking in-group favoritism to online prosocial behavior. Strong in-group favoritism reflects a high degree of group identification, which motivates individuals to direct helping behaviors toward in-group members in online spaces (Meng et al., 2022).

In recent years, this conclusion has been further consolidated and validated across a wider range of contexts: The salience of group identity in online spaces is a key factor influencing prosocial behavior: when group identity is clear and activated (Zheng et al., 2021), individuals' in-group preference significantly enhances their online prosocial behavior (Bai et al., 2025). In social media environments, users' identification with interest-based online communities (e.g., environmental groups, gaming forums) significantly predicts their engagement in collaborative and resource-sharing behaviors within those groups (Crockett et al., 2023). Similarly, research on online fundraising platforms demonstrates that donors' identification with a beneficiary group is a powerful predictor of their decision to contribute (Ryu et al., 2020).

In conclusion, helping in-group members is a fundamental mechanism for affirming one's group identity (Mokos et al., 2023). The architecture of the online world, by making group membership salient, transcending physical limits, and rendering helping behaviors publicly visible (Garcia and Rimé, 2019; Bosancianu et al., 2013), powerfully facilitates and strengthens the direct relationship between in-group favoritism and online prosocial behavior.

Based on this rationale, we propose the following hypothesis:

Hypothesis 1 (H1): In-group favoritism will positively predict online prosocial behavior among university students.

The Influence of In-group Preference on College Students‘ Online Prosocial Behavior: The Mediating Role of the Moral Self. Beyond its direct effect, the influence of in-group preference on college students' online prosocial behavior may also be realized indirectly through the activation of an individual's moral self-regulation process. Before exploring this mechanism, it is necessary to clarify the relationship between the “moral self” and “moral identity.” “Moral identity” typically refers to the extent to which moral traits (e.g., kindness, honesty) are integrated into an individual's self-concept (Hardy et al., 2022). It is a relatively stable trait that reflects how one views oneself (Aquino and Reed, 2002). In contrast, the core mediating variable of this study—the “moral self”—emphasizes a dynamic, state-like self-evaluative system. According to moral self-regulation theory, an individual's moral self is not static but functions as a “working self-concept” that is activated, monitored, and regulated within specific situations (Carnes and Lickel, 2018; Monin and Jordan, 2009). It evaluates the self after an action (e.g., “Doing this makes me feel like a good person”) (Aquino et al., 2021), thereby driving subsequent moral behavior to maintain a positive self-image (Hardy et al., 2022).

Based on this distinction, the present study posits that in-group preference activates this dynamic moral self-regulation process, rather than relying solely on an individual's inherent, stable moral identity. Specifically, when college students strongly identify with their group, they internalize the group's moral norms (e.g., mutual aid, altruism) (Zhang and Zhang, 2023; Zhang et al., 2023). At this point, these norms act as a “moral standard,” activating the individual's moral self-system (Mulder and Aquino, 2020). The moral self then compares its current self-image against this standard, generating a motivation to “confirm” or “enhance” one's moral self-worth through altruistic actions (Zhong et al., 2009). Consequently, performing prosocial behavior within an in-group context becomes a strategic action to actively regulate the moral self. By helping “one of us,” (Wang et al., 2023) individuals not only satisfy social norms but also gain immediate self-integrity confirmation of being “a moral person” (van der Lee et al., 2021). The unique features of the online environment—the public visibility of actions and the immediacy of feedback—significantly amplify this regulatory effect. The “moral payoff” of prosocial behavior becomes readily accessible, allowing individuals to efficiently complete a cycle of “activation-action-confirmation” in moral self-regulation (Zhao et al., 2020; Wellman and Gulia, 2018).

Substantial empirical research supports this pathway based on dynamic self-regulation. For instance, after recalling their own immoral behavior, individuals exhibit stronger prosocial tendencies to repair their damaged moral self-image (Sachdeva et al., 2009). This mechanism also applies at the group level: when group identity is activated, individuals are more inclined to engage in prosocial behavior to uphold the collective's moral image, thereby repairing or enhancing self-regard closely tied to the group (Barkan et al., 2012). Specific to online contexts, Zhang et al. (2025) and Chen et al. (2021) revealed a “public effect” of moral self-regulation, where social identity drives prosocial behavior by influencing individuals' moral self-perception. Similarly, Research by Chell et al. (2021) on online fundraising also demonstrated that the perceived “opportunity to enhance one's moral self-image” is a key predictor of donation intention. These studies collectively validate the theoretical value of conceptualizing the moral self as a dynamic construct that can be situationally modulated.

It is noteworthy that this regulatory process can be influenced by the type of group. In voluntary, affectively-based groups (e.g., charity clubs), individuals form stronger moral bonds with the group (Zhang and Zhang, 2023), and their motivation to maintain the moral self as a “good member” through actions is significantly stronger than in prescribed groups (Hertz and Krettenauer, 2016). Research by Yoshida et al. (2020) on university “learning communities” provides an example: students sharing materials to help “their own” is not merely altruism but a dynamic process of constructing and confirming their moral self as “a good, sharing community member.” Within the Chinese cultural context, collectivist values reinforce the symbiotic relationship between personal morality and collective honor (Sang, 2023). This imbues the act of helping in-group members with deeper cultural script significance (Li and Hu, 2025). By fulfilling the moral duty of acting for “us,” individuals' moral selves gain stronger cultural affirmation and psychological motivation (Pastor et al., 2024).

In summary, for Chinese college students who value collective honor, the pathway through which in-group preference influences online prosocial behavior—by activating a state-like moral self (rather than relying solely on trait-like moral identity)—possesses significant psychological realism and cultural congruence. Therefore, we propose Hypothesis 2: The moral self mediates the relationship between in-group preference and online prosocial behavior.

As an extension of offline prosocial behavior into the digital sphere, online prosocial behavior serves as a vital force for maintaining harmony within online communities. Although in-group preference is considered a key driving factor, its underlying mechanisms are complex (Huang and Xin, 2024). Critically, in-group preference does not always automatically activate a positive moral self; it can also lead to intergroup bias and exclusion (Xue et al., 2013; Leach, 2010). Therefore, investigating the conditions under which in-group preference positively predicts the moral self is central to delineating the boundaries of its positive effects.

Belief in a just world may play a crucial moderating role in this context. According to Belief in a just world theory, individuals with a strong Belief in a just world possess a fundamental motivation to believe that the world they live in is just and that people get what they deserve (Dalbert, 2001; Hafer and Sutton, 2016). This belief is not merely a cognitive schema but also a psychological resource that drives behavior (Hou and Wang, 2019): individuals high in Belief in a just world tend to act in ways that maintain and validate the justice of the world (Lerner, 1980). Based on self-consistency theory, individuals need to ensure their actions align with the core belief that “the world is just” (Xu Y. Y. et al., 2024; Hafer and Sutton, 2016). Examining this logic within the framework of moral self-regulation provides a clearer understanding of the moderating mechanism of Belief in a just world.

As previously discussed, the process by which in-group preference activates the moral self is essentially a dynamic process wherein individuals internalize group norms and evaluate themselves accordingly (Christner et al., 2022). The level of Belief in a just world profoundly influences the direction and intensity of this “internalization-evaluation” process. Specifically, for individuals high in Belief in a just world, when confronted with their in-group, their preference does not remain merely at the level of group belonging or favoritism. Instead, it is channeled into actions that embody and practice universal moral principles such as “justice” and “mutual aid.” (Xu L. et al., 2024; Bartholomaeus and Strelan, 2019). In other words, when high-Belief in a just world individuals help in-group members, they do not just see it as “helping us”; they interpret it as “doing the right thing”—by supporting the wellbeing of their in-group, they are effectively practicing and reinforcing their core belief in a just world (Correia et al., 2021). This interpretive process of “moralizing” group preference endows in-group preference with stronger moral significance, thereby significantly enhancing its activation of the moral self-concept. When individuals realize, “I help my in-group members because a just world demands it,” the positive feedback effect of this action on the moral self is amplified, leading to a stronger sense of self-affirmation as “a moral person.”

Empirical research supports this perspective. For instance, Liu (2023) found that adolescents with a Belief in a behaviors less constrained by group boundaries, suggesting their prosocial motivation was more morally universal. In other words, a strong Belief in a just world makes it easier for their prosocial actions to be internalized as components of the moral self. Research by Zhang et al. (2025) and Zhao et al. (2018) also suggests that individuals with a strong belief in a just world are more inclined to establish moral causal links in their behavior, a core feature of the moral self-regulation process.

Therefore, Belief in a just world serves as a crucial boundary condition for understanding how in-group preference influences online prosocial behavior through the moral self. A strong Belief in a just world prompts individuals to interpret their in-group preference as a moral act of upholding justice, thereby significantly strengthening the positive predictive effect of in-group preference on the moral self. Accordingly, we propose Hypothesis 3: Belief in a just world moderates the mediating model of in-group preference on online prosocial behavior via the moral self, specifically moderating the first stage of this mediating path (i.e., in-group preference → moral self).

To summarize, this study proposes the following three hypotheses:

Hypothesis 1 (H1): In-group favoritism will positively predict online prosocial behavior.

Hypothesis 2 (H2): The moral self will mediate the relationship between in-group favoritism and online prosocial behavior.

Hypothesis 3 (H3): Belief in a just world will moderate the relationship between in-group favoritism and the moral self.

**Figure 1:**
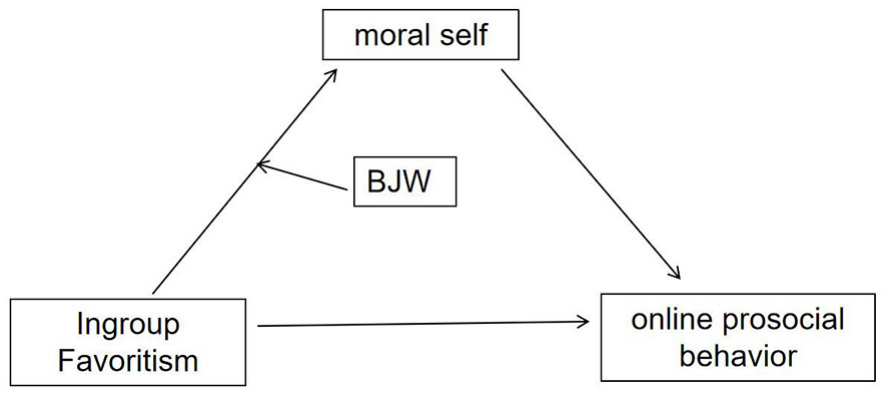
The moderated mediation model.

The conceptual framework of this study is illustrated in Figure 1.

## 2 Materials and methods

To ensure the validity of the experimental materials, we first conducted a pilot study. Following this, the main experiment employed a behavioral priming paradigm to investigate the causal relationships between the core variables. After the experiment, data were processed and analyzed using SPSS 26.0 to test the proposed hypotheses.

## 3 Pilot study: priming and measuring belief in a just world

### 3.1 Aims of the pilot study

This pilot study aimed to validate the effectiveness of different materials in experimentally priming Belief in a Just World (BJW). Specifically, the objectives were:

To activate a high BJW state in participants using an experimental paradigm adapted from Liang et al. (2016).

To activate a low BJW level in participants using popular science materials.

To measure the difference in BJW between the two groups using the Chinese version of the General Belief in a Just World Scale (GBJWS) translated and adapted by Su et al. (2012), thereby testing the effectiveness of the experimental manipulation and providing a validated paradigm for the subsequent main study.

### 3.2 Method

#### 3.2.1 Participants

This pilot study planned to recruit 60 participants. The electronic questionnaire was distributed via the Credamo platform, and data collection adhered to the principle of voluntary participation. Upon completion of the study, a small gift was provided to each participant as a token of appreciation for their time.

#### 3.2.2 Experimental materials

(1) Priming Group: High BJW Activation Materials

Materials were presented via a Word document. The text described a scenario in which an elderly person was hit and injured by a truck on a national highway, and the driver fled the scene. A passerby, Mr. Chen, discovered the unconscious victim and promptly took him to the hospital, saving his life. The family expressed deep gratitude to Mr. Chen. A week later, with the help of traffic camera footage and the victim's account, the authorities apprehended the hit-and-run driver. The driver was subsequently sentenced to 1 year in prison and ordered to compensate the victim's family.

(2) Control Group: Low BJW Activation Materials

Materials were presented via a Word document. The text contained neutral content about the solar system, stating: “There are eight planets in the solar system. From the innermost to the outermost, their orbits are Mercury, Venus, Earth, Mars, Jupiter, Saturn, Uranus, and Neptune. The scientific community currently hypothesizes the existence of a ninth planet in the outer solar system, which might be a super-Earth. Its gravitational effects could explain the peculiar clustering of orbits observed in a group of extreme trans-Neptunian objects.”

(3) General Belief in a Just World Scale (GBJWS)

The Chinese version of the General Belief in a Just World Scale (GBJWS), translated and adapted by Su et al. (2012), was used. This scale consists of 6 items rated on a 6-point Likert scale (1 = “Strongly Disagree” to 6 = “Strongly Agree”). The BJW score was calculated as the mean of the 6 items, with higher scores indicating a stronger belief in a just world. In this pilot study, the Cronbach's α for the GBJWS was.85.

### 3.3 Procedure

Upon entering the experiment, participants were randomly assigned to either the priming group or the control group, with 30 participants in each group. After reading the assigned priming material, all participants completed the General Belief in a Just World Scale.

### 3.4 Results

An independent samples *t*-test was conducted to compare the scores on the GBJWS between the priming group and the control group. The results revealed that the priming group (*M* = 4.09, SD = 0.41) scored significantly higher than the control group (*M* = 2.94, SD = 0.60), *t* (58) = −8.45, *p* < .001.

### 3.5 Conclusion

The experimental manipulation (High BJW priming vs. Low BJW control) had a significant effect on participants' belief in a just world. This indicates that the priming materials were effective and successfully induced different levels of BJW.

## 4 Main experiment

### 4.1 Aims

Building upon the pilot study, the main experiment aimed to investigate the influence of in-group favoritism on online prosocial behavior, as well as the mediating role of the moral self and the moderating role of belief in a just world (BJW).

### 4.2 Participants

The required sample size was calculated using G^*^Power 3.1. For a 2 (In-group Favoritism: high vs. low) × 2 (BJW: primed vs. control) between-subjects analysis of variance (ANOVA), with parameters set to a medium effect size (*f* = 0.35), a significance level of α = 0.05, and a statistical power of 1 – β = 0.85, the calculation indicated a minimum total sample size of 112 participants (28 per cell).

A total of 185 university students were recruited to participate in the experiment. After data screening, 42 invalid responses were excluded based on the following criteria: (1) completion time of less than 5 min; (2) patterned responses (e.g., selecting the same option for more than 70% of consecutive items); and (3) missing responses to any item. The final valid sample consisted of 143 participants (71 males, 72 females), with a mean age of 19.32 years. The experimental conditions were balanced as follows: 40 participants in the Low In-group Preference-Belief in a Just World Control group, 33 in the Low In-group Preference-Belief in a Just World Priming group, 32 in the High In-group Preference-Belief in a Just World Control group, and 36 in the High In-group Preference-Belief in a Just World Priming group. All procedures performed in this study were in accordance with the ethical standards of the institutional and/or national research committee and with the 1,964 Helsinki declaration and its later amendments. Informed consent was obtained from all individual participants included in the study.

### 4.3 Materials and procedure

#### 4.3.1 Experimental materials

(1) In-group Favoritism Priming Videos

Given that all participants were recruited from University A in Anhui Province, the promotional videos of University A and University B (another university in Anhui Province) were used as the experimental materials to prime high and low in-group preference, respectively.

(2) In-group Favoritism Scale (Leach et al., 2008)

The level of in-group favoritism was measured using the In-group Identification Scale developed by Leach et al. (2008). This scale consists of 14 items, each rated on a 5-point Likert scale from 1 (“Strongly Disagree”) to 5 (“Strongly Agree”). Higher total scores indicate a stronger level of in-group favoritism. In this study, the scale demonstrated high internal consistency, with a Cronbach's α of.90.

(3) Belief in a Just World Scale

The personal belief in a just world was measured using the Belief in a Just World Scale (BJW) developed by Dalbert (1999) and revised by Su et al. (2012). This scale comprises 13 items rated on a (“Completely Inconsistent”) to 5 (“Completely Consistent”). Higher total scores reflect a stronger personal belief in a just world. In the present study, the scale's Cronbach's α was.88.

(4) Moral Self Subscale

The moral self subscale from the Tennessee Self-Concept Scale (TSCS; Fitts, 1965) was employed to measure the moral self. This rated on a 5-point Likert scale from 1 (“Completely Inconsistent”) to 5 (“Completely Consistent”). Higher scores indicate a higher level of moral self-concept. In this study, the subscale showed acceptable internal consistency, with a Cronbach's α of.71.

(5) Measurement and Operationalization of Online Prosocial Behavior

**Figure 2:**
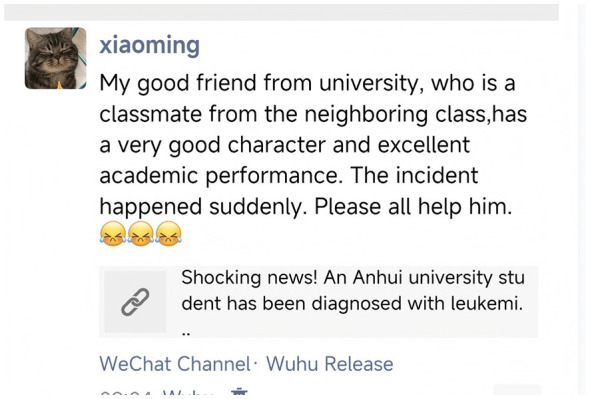
Experimental scenario (a): Online donation context.

**Figure 3:**
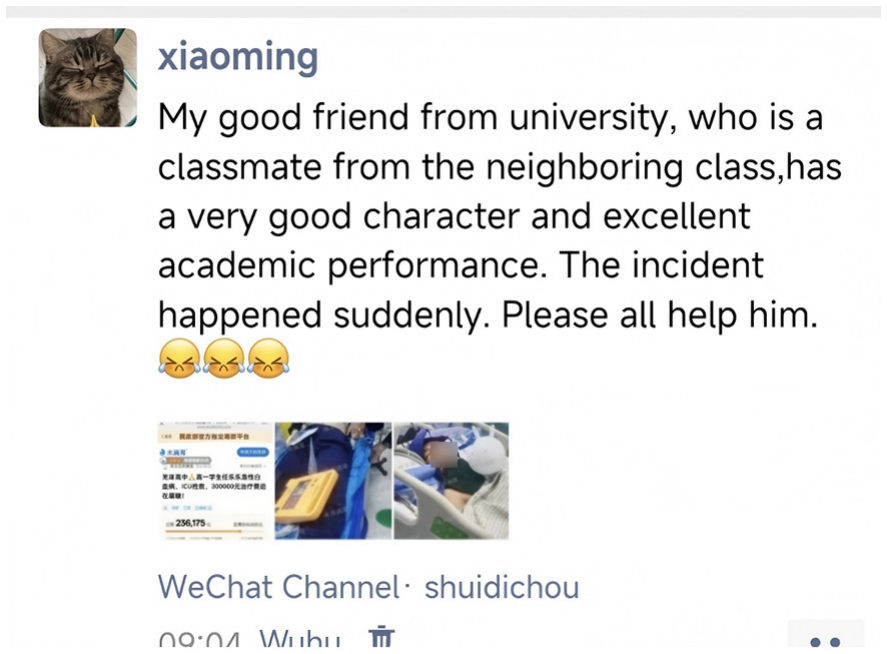
Experimental scenario (b): Online donation context.

This measure was adapted from Wang and Tong's (2015) research. Participants were presented with an online donation scenario (see Figures 2, 3) in which they read a social media post about a friend's serious illness. The text description read: “Xiao Ming is a fellow student you added as a contact after a campus event. The image below shows a post from his social media feed. His friend has been suddenly diagnosed with a serious illness, and the family is struggling to afford the medical treatment. Please read the scenario above and type your donation intention and a comment in the text box below. For every 10 characters in your comment, we will donate 10 RMB to the person in the post; 20 characters or more will trigger a 20 RMB donation; 30 characters or more, a 50 RMB donation; and 50 characters or more, a 100 RMB donation. The content of your comment will serve as important data for this research analysis.”

In this study, the number of words in participants‘ comments under the hypothetical donation scenario was used as the quantitative indicator of online prosocial behavior. This operationalization is grounded in the following theoretical considerations. First, in anonymous online donation contexts, where actual donating behavior cannot be tracked, comment length can be viewed as a behavioral indicator of an individual's cognitive effort and empathic engagement. A higher word count typically reflects greater investment of cognitive resources in organizing language and expressing concern, suggesting stronger prosocial motivation (Pfattheicher et al., 2022; Eisenberg et al., 2010). Second, word count can also be interpreted as a form of moral signaling. In public or semi-public online social spaces, composing longer and more thoughtful comments serves as a social signal to others—including the researcher, the recipient, and bystanders—that “I am a moral and empathetic person” (Jordan and Rand, 2021). Third, previous studies have adopted similar paradigms, using the amount of linguistic output in online contexts (e.g., comment length, detail of responses) as a valid indicator of prosocial intention and engagement (Wang et al., 2025; Li et al., 2025). Therefore, the word count indicator in this study is intended to capture individuals' expressive prosocial behavior in a specific online context, rather than directly measuring material assistance (e.g., actual monetary donations). This distinction will be explicitly acknowledged in the interpretation of the findings.

#### 4.3.2 Experimental procedure

This study employed a 2 (In-group Preference: high vs. low) × 2 (Belief in a Just World: priming vs. control) between-subjects experimental design to examine the effects of different levels of in-group preference and belief in a just world on the moral self and online prosocial behavior. The dependent variable was the level of prosocial behavior exhibited by participants in an online context, operationalized as the number of words in their comments. To control for potential confounding effects of demographic variables on online prosocial behavior (Li et al., 2025; Zhao, 2018), gender, grade, and place of origin variables in subsequent analyses.

The specific procedure is illustrated in Figure 3. First, participants were randomly assigned to one of the four experimental groups. Participants in each group began by watching a video clip designed to induce either a high or low level of in-group favoritism. They then completed the In-group Favoritism Scale to check the validity of the video manipulation and assess their current level of in-group favoritism.

Next, participants in the control group read a neutral text about the solar system (the same text used in the pilot study), while those in the priming group read the text describing the “Mr. Chen rescue incident” (also from the pilot study) to activate their Belief in a Just World.

Subsequently, all participants completed the Belief in a Just World Scale and the Moral Self Subscale to measure the moderator and mediator variables, respectively.

Finally, all participants were presented with the hypothetical social media post about a donation appeal. They were instructed to type a comment based on their personal donation intention. The word count of their comment served as the quantitative measure of their online prosocial behavior.

Upon completion of the experiment, all participants were thanked for their support and received a small gift. To address potential ethical concerns or psychological discomfort that the experimental materials might have elicited, the experimenter debriefed all participants afterward, explaining that all materials were fictional and used solely for scientific research purposes to mitigate any potential negative effects.

### 4.4 Data processing

Data were analyzed using SPSS 26.0 and the PROCESS macro for SPSS (Version 4.1) developed by Hayes (2017), employing Model 4 and Model 7.

### 4.5 Results

#### 4.5.1 Manipulation checks

Check for In-group Favoritism Manipulation:

An independent samples *t*-test was conducted to compare the In-group Favoritism Scale scores between the priming group and the control group. The results indicated that the priming group (*M* = 5.54, SD = 0.63) scored significantly higher than the control group (*M* = 4.84, SD = 0.50), *t* (141) = −7.324, *p* < 0.001, Cohen's *d* = 1.23. This result confirms that the experimental manipulation of in-group favoritism was effective and produced a large difference between the groups.

Check for Belief in a Just World (BJW) Manipulation:

An independent samples *t*-test was conducted to compare the BJW scores between the priming group and the control group. Levene's test indicated unequal variances (*F* = 15.825, *p* < 0.001), so the corrected results were adopted. The analysis revealed that the priming group (*M* = 3.91, SD = 0.66) scored significantly higher than the control group (*M* = 3.72, SD = 0.45), *t* (123.58) = −2.063, *p* = .041, Cohen's *d* = 0.35. This result confirms the effectiveness of the BJW manipulation.

#### 4.5.2 Effects of in-group favoritism and BJW on online prosocial behavior

A two-way between-subjects analysis of variance (ANOVA) was performed with online prosocial behavior (comment word count) as the dependent variable and In-group Favoritism (high/low) and BJW (primed/control) as independent factors.

The results revealed a significant main effect of in-group favoritism, [*F*_(1.139)_ = 4.34, *p* = 0.039, η*p*^2^ = 0.03]. The high in-group favoritism group (*M* = 4.37, SD = 0.12) exhibited a significantly higher level of online prosocial behavior than the low in-group favoritism group (*M* = 4.03, SD = 0.12).

A significant main effect of BJW was also found, [*F*_(1.139)_ = 4.53, *p* = 0.036, η*p*^2^ = 0.03. The BJW-primed group (*M* = 4.38, SD = 0.12) showed a significantly higher level of online prosocial behavior than the control group (*M* = 4.03, SD = 0.12).

Crucially, the interaction between in-group favoritism and BJW was significant, [*F*_(1.139)_ = 7.50, *p* = 0.007, η*p*^2^ = 0.05].

Following the significant interaction, simple effects analyses were conducted to probe the nature of this interaction. The results showed that for participants in the BJW-primed condition, the main effect of in-group favoritism was significant, [*F*_(1.139)_ = 11.65, *p* =.001]. *Post-hoc* comparisons revealed that within the BJW-primed condition, individuals with high in-group favoritism (*M* = 4.43, SE = 0.16) engaged in significantly more online prosocial behavior than those with low in-group favoritism (*M* = 3.63, SE = 0.18). However, for participants in the control condition, the main effect of in-group favoritism was not significant, [*F*_(1.139)_ = 0.22, *p* = 0.642, with no significant difference in online prosocial behavior between the high (*M* = 4.32, SE = 0.17) and low (*M* = 4.43, SE = 0.16) in-group favoritism groups. This interaction pattern suggests that the priming of belief in a just world may moderate the effect of in-group preference on online prosocial behavior: when individuals' belief in a just world is situationally activated, in-group preference has a significant positive predictive effect on their online prosocial behavior.

#### 4.5.3 Mediation effect analysis

(1) Correlational analyses among the key variables

The results of the correlational analyses among the key variables are presented in Table 1. In-group favoritism was positively correlated with the moral self, belief in a just world, and online prosocial behavior, with all correlations being statistically significant.

**Table 1:** Correlation analysis among the variables.

**Variables**	**1**	**2**	**3**	**4**
1 Group preferences	1			
2 Moral self	0.41^**^	1		
3 Belief in a just world	0.49^**^	0.39^**^	1	
4 Online prosocial behavior	0.28^**^	0.32^**^	0.22^**^	1

^*^p < 0.05, ^**^p < 0.01, ^***^p < 0.001.

(2) Testing the mediating role of the moral self

To examine whether the moral self mediates the relationship between in-group favoritism and online prosocial behavior, a mediation analysis was conducted using Model 4 from the PROCESS macro (Hayes, 2017). The results are summarized as shown in Tables 2 and 3.

**Table 2:** Regression analysis results of the mediation model.

**Result variable**	**Predictor variable**	**β**	**SE**	***t***	**95% CI**
Moral self	Constant term	0.10	0.08	1.28	[−0.06,0.26]
	Group preferences	0.46	0.09	5.33^***^	[0.29,0.62]
Online prosocial behavior	Constant term	−0.08	0.09	−0.97	[−0.26,0.09]
	Group preferences	0.22	0.10	2.13^*^	[0.02,0.42]
	Moral self	0.26	0.09	2.80^*^	[0.08,0.44]

^*^p < 0.05, ^**^p < 0.01, ^***^p < 0.001.

**Table 3:** Decomposition table of total effects, direct effects and mediating effects.

**Effect path**	**Effect value**	**Boot SE**	**Boot 95% CI**	**Standardization effect**
Overall effect (c)	0.34^**^	0.10	[0.15, 0.53]	0.28
Direct effect (c')	0.22^*^	0.10	[0.02, 0.42]	0.18
Indirect effect (a × b)	0.12^*^	0.05	[0.03, 0.23]	0.10

^*^p < 0.05, ^**^p < 0.01, ^***^p < 0.001.

The analysis indicated that the moral self plays a partial mediating role in the relationship between in-group favoritism and online prosocial behavior. In-group favoritism not only had a direct positive effect on online prosocial behavior (β = 0.22, ^*^p^*^ = 0.040) but also exerted an indirect effect through the moral self (indirect effect = 0.12, 95% CI [0.03, 0.23]).

This indirect effect accounted for 34.90% of the total effect, indicating that the moral self is a significant mechanism explaining how in-group favoritism influences online prosocial behavior.

#### 4.5.4 Moderated mediation analysis

A moderated mediation analysis was conducted using Model 7 from the PROCESS macro (Hayes, 2018). The model specified in-group favoritism as the independent variable, the moral self as the mediator, online prosocial behavior as the dependent variable, and belief in a just world (BJW) as the moderator. Gender, grade, and place of origin were included as covariates.

The results (see Table 4) revealed a significant moderated mediation effect. Specifically, BJW significantly moderated the first stage of the mediation path—the relationship between in-group favoritism and the moral self (β = 0.38, ^*^p^*^ < 0.001). The index of moderated mediation was 0.10, with a 95% confidence interval of [0.03, 0.19], which does not include zero, further supporting the significance of the moderated mediation effect.

**Table 4:** Moderated mediation model.

**Predictor variable**	**Equation1**			**Equation2**		
	**Dependent variable:Moral self (** ***M*** **)**			**Dependent variable: network prosocial behavior (** ***Y*** **)**		
	β	**SE**	***t***	β	**SE**	***t***
Constant term	−0.15^*^	0.08	−1.99	−0.13	0.08	−1.61
Intra-group preference (X)	0.19^*^	0.10	2.00	0.22^*^	0.10	2.13
Belief in a just world (W)	0.23^**^	0.09	2.67	−0	–	–
X × W	0.38^***^	0.09	4.37	–	–	–
Moral self (M)	–	–	–	0.26^**^	0.10	2.80
**Model indicators**						
R^2^	0.31			0.13		
F	20.77^***^			10.38^***^		
ΔR^2^	0.10^***^					

^*^p < 0.05, ^**^p < 0.01, ^***^p < 0.001.

**Figure 4:**
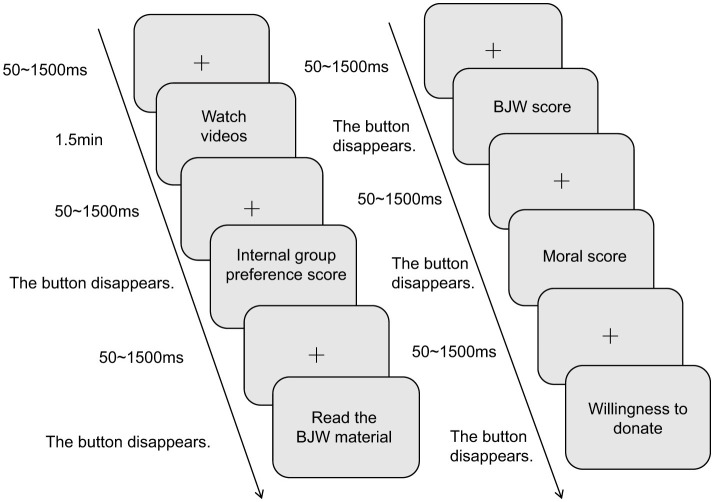
Experimental procedure flowchart.

Simple slope analysis was performed to probe this interaction. As illustrated in Figure 4, the indirect effect of in-group favoritism on online prosocial behavior via the moral self was significant for individuals with a high level of BJW (+1 SD; effect = 0.14, 95% CI [0.04, 0.26]). In contrast, the indirect effect was not significant for those with a low level of BJW (−1 SD; effect = 0.04, 95% CI [−0.05, 0.13]).

These results indicate that belief in a just world strengthens the indirect effect of in-group favoritism on online prosocial behavior through the moral self. The mediating role of the moral self is significant only when individuals hold a stronger belief in a just world.

## 5 General discussion

Through a moderated mediation model, this study systematically investigated the synergistic mechanisms by which in-group favoritism, belief in a just world (BJW), and the moral self influence online prosocial behavior.

### 5.1 The direct effect of in-group favoritism on online prosocial behavior

First, the present study reaffirmed the significant positive predictive effect of in-group preference on online prosocial behavior, providing new evidence for the applicability of Social Identity Theory (Tajfel and Turner, 1979) in the digital sphere. Although the effect size was small, this finding was statistically robust, suggesting that individuals' fundamental motivation to engage in positive behavior through group categorization persists even in digital media. For instance, Wang et al. (2022); Wang and Meng (2022) found that college students' identification with their university significantly predicted their online prosocial behavior. Similarly, Chen and Shi (2019) indicated that donors' preference for the group to which the help-seeker belongs is a key factor facilitating donation decisions through the mediation of empathy. Even in relatively unique online spaces such as online gaming environments—which can easily induce negative behaviors—when players' sense of belonging to a team or guild is activated, their malicious behaviors significantly decrease (Beres et al., 2021). Collectively, this evidence suggests that individuals' fundamental motivation to gain positive self-esteem and a sense of belonging through group categorization remains unchanged despite the digitization of media. Online interaction does not dissolve group boundaries (Levine and Cunningham, 2019); instead, it provides a new behavioral arena for the expression of in-group preference.

### 5.2 The mediating role of the moral self

Furthermore, this study found that the moral self mediates the relationship between in-group preference and online prosocial behavior. This result is not a simple replication of previous research but rather a more refined theoretical positioning of the mediating mechanism. Prior studies have often employed “moral identity”—a relatively stable trait-like construct—to explain similar pathways (Liu, 2023; Kou, 2020; Aquino and Reed, 2002), suggesting that group norms are internalized as part of an individual's stable self-concept, which in turn drives behavior.

However, drawing on Moral Self-Regulation Theory (Monin and Jordan, 2009), the present study introduces the state-like alternative dynamic process: In-group preference does not directly shape a more stable “good person” trait (van Zomeren and Louis, 2019); rather, it situationally activates a heightened self-evaluation of “being a moral person at this moment.” When college students watched their university's promotional video, their group identity was awakened, which immediately triggered moral scripts tied to their group identity (e.g., “People from our school should help each other”). At this point, the moral dimension of the individual's working self-concept becomes highly salient. To confirm or enhance this activated positive moral self-image, individuals subsequently generate motivation to “prove” themselves through prosocial behavior (Yan and Hollingshead, 2022). Social Intuitionist Theory (Haidt, 2001) provides a processual explanation for this: Strong group preference makes “helping one's own” a rapid moral intuition, and subsequent prosocial behavior (e.g., posting supportive comments) is a rationalized enactment of this intuition, aimed in part at maintaining moral self-consistency after the action. Therefore, the contribution of this study lies in extending the mediating mechanism from “who I am” (stable moral identity) to “how I feel right now” (dynamic moral self-regulation) (Hardy and Carlo, 2011), offering a possible explanatory pathway for understanding situational prosocial behavior in online contexts.

### 5.3 The moderating role of belief in a just world

Another core finding of this study is that Belief in a Just World (BJW) significantly moderated the “in-group preference → moral self” pathway. This result not only identifies an important boundary condition of the model but also deepens our understanding of the social function of BJW (Tang et al., 2025). Unlike previous studies that treated BJW merely as a buffering or enhancing variable (Huang and Xin, 2024; Correia and Sutton, 2021), the present study proposes an explanatory framework of “moralization conversion” based on Cognitive Dissonance Theory (Festinger, 1957).

For individuals high in BJW, “the world is just” is a core cognitive commitment. When in-group preference is activated, this tendency toward pure “favoring one's own” may create subtle cognitive conflict with their belief that “everyone should get what they deserve” (Hafer et al., 2020; Hafer and Bègue, 2005). To alleviate this potential dissonance (Harmon-Jones and Mills, 2019), high-BJW individuals initiate a process of cognitive reconstruction: they reinterpret what might otherwise be viewed as “partiality” as an act of upholding justice. For example, helping in-group members is no longer merely “helping one's own,” but is interpreted as “supporting our rule-abiding, excellent group, which is itself an embodiment of justice” (Xu and Han, 2024). Through this “moralization” conversion of group preference, in-group preference may no longer conflict with BJW but instead becomes empirical evidence supporting their belief in a just world. This reconstructive process endows in-group preference with stronger moral significance, thereby potentially strengthening its positive activation effect on the moral self.

## 6 Limitations and future directions

This study has several limitations. First, regarding the measurement of online prosocial behavior. This study used comment word count in a hypothetical scenario as an indicator, which measures expressive prosocial behavior rather than actual donating behavior and cannot capture the quality dimension of prosocial behavior. Although word count can theoretically reflect cognitive effort and empathic engagement, this indicator remains an indirect measure. Future research could combine content analysis or behavioral tracking techniques (e.g., real donation options) to more comprehensively capture this construct. Moreover, the ecological validity of hypothetical scenarios is limited; subsequent studies could conduct field experiments on real online platforms for validation. Second, the manipulation intensity of Belief in a Just World. This study employed a single-session text priming paradigm, which may have triggered only transient situational BJW. Caution is needed when generalizing the conclusions to stable trait-like BJW. Future research could adopt multi-session priming paradigms or directly measure trait BJW to examine the cross-level stability of the model. Third, the homogeneity of the sample. The participants in this study were all university students. While this helps control for variables such as age and education level, it also limits the generalizability of the findings to other populations. Future research should test the model's applicability in more diverse samples.

## 7 Conclusion

Based on the moderated mediation analysis, this study concludes that:

(1) In-group favoritism significantly and positively predicts online prosocial behavior.

(2) The moral self plays a partial mediating role in the relationship between in-group favoritism and online prosocial behavior.

(3) Belief in a just world moderates the first stage of the mediation path, specifically the effect of in-group favoritism on the moral self.

## Data Availability

The datasets presented in this article are not readily available because the data contains sensitive information that could compromise the privacy of research participants, and the data are part of an ongoing research project. Requests to access the datasets should be directed to Qingsong Sang, 7210qs1@ahnu.edu.cn.
